# Detection of prostate cancer bone metastases with fast whole-body ^99m^Tc-HMDP SPECT/CT using a general-purpose CZT system

**DOI:** 10.1186/s40658-022-00517-4

**Published:** 2022-12-12

**Authors:** Samuli Arvola, Marko Seppänen, Kirsi L. Timonen, Pentti Rautio, Otto Ettala, Mikael Anttinen, Peter J. Boström, Tommi Noponen

**Affiliations:** 1 Department of Clinical Physiology, Nuclear Medicine and Turku PET Centre, Turku University Hospital and University of Turku, Kiinamyllynkatu 4-8, P.O. Box 52, 20521 Turku, Finland; 2 Department of Clinical Physiology and Nuclear Medicine, Hospital Nova of Central Finland, Jyväskylä, Finland; 3 Department of Clinical Physiology, North Karelia Central Hospital, Joensuu, Finland; 4 Department of Urology, University of Turku and Turku University Hospital, Turku, Finland; 5 Department of Medical Physics, Turku University Hospital, Turku, Finland

**Keywords:** Whole-body, Bone, SPECT/CT, Time reduction, CZT

## Abstract

**Background:**

We evaluated the effects of acquisition time, energy window width, and matrix size on the image quality, quantitation, and diagnostic performance of whole-body ^99m^Tc-HMDP SPECT/CT in the primary metastasis staging of prostate cancer.

**Methods:**

Thirty prostate cancer patients underwent ^99m^Tc-HMDP SPECT/CT from the top of the head to the mid-thigh using a Discovery NM/CT 670 CZT system with list-mode acquisition, 50-min acquisition time, 15% energy window width, and 128 × 128 matrix size. The acquired list-mode data were resampled to produce data sets with shorter acquisition times of 41, 38, 32, 26, 20, and 16 min, narrower energy windows of 10, 8, 6, and 4%, and a larger matrix size of 256 × 256. Images were qualitatively evaluated by three experienced nuclear medicine physicians and quantitatively evaluated by noise, lesion contrast and SUV measurements. Diagnostic performance was evaluated from the readings of two experienced nuclear medicine physicians in terms of patient-, region-, and lesion-level sensitivity and specificity.

**Results:**

The originally acquired images had the best qualitative image quality and lowest noise. However, the acquisition time could be reduced to 38 min, the energy window narrowed to 8%, and the matrix size increased to 256 × 256 with still acceptable qualitative image quality. Lesion contrast and SUVs were not affected by changes in acquisition parameters. Acquisition time reduction had no effect on the diagnostic performance, as sensitivity, specificity, accuracy, and area under the receiver-operating characteristic curve were not significantly different between the 50-min and reduced acquisition time images. The average patient-level sensitivities of the two readers were 88, 92, 100, and 96% for the 50-, 32-, 26-, and 16-min images, respectively, and the corresponding specificities were 78, 84, 84, and 78%. The average region-level sensitivities of the two readers were 55, 58, 59, and 56% for the 50-, 32-, 26-, and 16-min images, respectively, and the corresponding specificities were 95, 98, 96, and 95%. The number of equivocal lesions tended to increase as the acquisition time decreased.

**Conclusion:**

Whole-body ^99m^Tc-HMDP SPECT/CT can be acquired using a general-purpose CZT system in less than 20 min without any loss in diagnostic performance in metastasis staging of high-risk prostate cancer patients.

**Supplementary Information:**

The online version contains supplementary material available at 10.1186/s40658-022-00517-4.

## Introduction

Whole-body bone SPECT/CT is a more accurate method than planar bone scintigraphy for the detection of bone metastases in cancer patients [1–6]. Currently, a separate CT examination is used to compensate for the low specificity of planar bone scintigraphy. Nonetheless, the diagnostic confidence obtained with SPECT/CT is higher than that of combined planar bone scintigraphy and CT [7, 8]. Despite these benefits, the current use of bone SPECT/CT is often limited to partial-body imaging as an addition to the routinely performed planar bone scintigraphy. This limitation is partly due to the lack of fast acquisition protocols for whole-body bone SPECT/CT [8].

The total acquisition time of a whole-body SPECT/CT performed according to the current guidelines is at least 40 min when the detector and bed movements are included [3, 4]. These guidelines were written prior to the advent of the general-purpose cadmium-zinc-telluride (CZT) system [9], which allows optimization of acquisition protocols, including imaging time. The properties of CZT detector-based SPECT systems enable imaging with higher sensitivity and spatial and energy resolution than systems based on conventional NaI detectors [10]. The higher sensitivity allows for faster acquisition or lower injected activity.

CZT-based SPECT systems acquire data in list-mode, which can be resampled into sinograms with different acquisition parameters. Shortening bone SPECT acquisitions using list-mode data from a CZT SPECT system have been previously introduced by Gregoire et al. [11]. However, the effect of a short acquisition time on the diagnostic performance of whole-body SPECT/CT has not been studied, as earlier research has mainly focused on visually evaluated image quality.

We explore the potential of the high spatial and energy resolution of the CZT detector on SPECT image quality by increasing the acquisition matrix and narrowing the energy window. The large matrix enhances spatial details in images and might improve the visibility of small lesions. Narrowing the energy window can be regarded as the optimal scatter correction method because the scattered photons are directly rejected in the preprocessing instead of being approximated and subtracted during the reconstruction [12]. These effects are studied by qualitative and quantitative image analyses, as well as by measuring standardized uptake values (SUVs) of lesions. The effect of post-filtering on the fast acquired SPECT images is also investigated.

We also evaluate the effects of the acquisition time of SPECT on the diagnostic performance of whole-body ^99m^Tc-HMDP SPECT/CT in the primary metastasis staging of prostate cancer. The findings are validated against multimodal reference data consisting of ^18^F-PSMA-1007 PET/CT, whole-body diffusion-weighted magnetic resonance, and follow-up images. Our analyses are based on fused SPECT/CT images as opposed to SPECT without CT in previous studies [11, 13].

## Materials and methods

### Patients

This study included 30 prostate cancer patients at high risk for bone metastases who had undergone ^99m^Tc-HMDP planar bone scintigraphy and SPECT/CT, ^18^F-PSMA-1007 PET/CT, contrast-enhanced CT, and 1.5-T whole-body diffusion-weighted magnetic resonance imaging within 14 days. These patients were retrospectively selected from the population recruited for a previous clinical trial (NCT03537391). Fifteen patients had bone metastases, and the other 15 had only benign findings according to the ^99m^Tc-HMDP SPECT/CT readings of that trial [14].

All procedures performed in human participants were in accordance with the ethical standards of the institutional research committee and with the 1964 Helsinki declaration and its later amendments or comparable ethical standards. Informed consent to participate was obtained from all individuals included in the study.

### SPECT/CT acquisition

The SPECT images were acquired 185 ± 17 (mean ± SD) min after intravenous injection of 693 ± 22 (mean ± SD) MBq of ^99m^Tc-HMDP using a Discovery NM/CT 670 CZT system (GE Healthcare, Haifa, Israel). The SPECT system includes digital CZT detectors. The images were acquired in list-mode with the following parameters: wide-energy high-resolution collimators, three bed positions from the top of the head to the mid-thigh, step-and-shoot, body contouring, 60 views (120 projections) over 360° with 13-s acquisition time per view, 15% energy window centered at 140 keV, 128 × 128 matrix, 4.4 × 4.4 mm pixel size, and 4.4-mm slice thickness. Low-dose CT images were acquired immediately after SPECT from the top of the head to the mid-thigh with modulated mAs (noise index 70), 120 kVp, 1.35 pitch, and 2.5-mm slice thickness.

The gamma camera was calibrated for activity concentration measurement by imaging a uniform Jaszczak phantom (Data Spectrum Corporation, Durham, NC, USA) without any inserts inside and filled with water and 131.1 MBq of ^99m^Tc-pertechnetate. The calibration image was acquired in list-mode with the same parameters as the patient images.

### Data processing for qualitative and quantitative image analyses

The SPECT data were reconstructed with HybridRecon-Oncology software (version 3.0, HERMES Medical Solutions AB, Stockholm, Sweden) using the ordered-subset expectation maximization algorithm with 6 iterations and 15 subsets and corrections for photon attenuation, scatter, and collimator response. Attenuation correction was based on the attenuation coefficient maps derived from the CT images. Scatter correction was performed with a Monte Carlo simulation using 10^6^ simulated photons and two scatter update iterations. The collimator response was corrected using a Gaussian diffusion model. The images were filtered using a Gaussian filter with 7-mm full width at half maximum (FWHM).

From the calibration image, a conversion factor to convert the reconstructed counts into units of activity concentration (Bq/ml) was calculated as the ratio between true activity and reconstructed counts in a homogeneous volume of interest (VOI). Voxel SUVs were then calculated using the equation

$$\mathrm{SUV}=\frac{cW}{A},$$

where *c* is the activity concentration (Bq/ml), *W* is the patient body weight (g) converted to volume (ml) assuming a density of 1 g/ml, and *A* is the injected activity (Bq) corrected for decay and syringe residual activity.

For the quantitative and qualitative analyses, ten more image data sets were generated. The acquired list-mode data were resampled using Lister software on a Xeleris 4 workstation (GE Healthcare, Haifa, Israel) to produce sinograms with either the energy window narrowed from 15 to 10, 8, 6, or 4%, the matrix size increased from 128 × 128 to 256 × 256, or the acquisition time per view reduced from 13 to 10, 9, 7, 5, or 3 s. The idle time caused by the detector and bed movements was 11 min. Therefore, the acquisition times of 13, 10, 9, 7, 5, and 3 s per view correspond to total acquisition times of 50, 41, 38, 32, 26, and 20 min, respectively. These data sets were reconstructed as the original SPECT data. The energy window narrowing was also applied to the calibration image, and separate conversion factors were calculated for the narrower energy windows.

### Data processing for diagnostic performance analysis

For the evaluation of diagnostic performance with different acquisition times, three additional image data sets with total acquisition times of 32, 26, and 16 min were generated. The dataset with 16-min total acquisition time was generated by halving the number of views from 60 to 30 in the images with 5-s acquisition time per view. The number of views was halved using Angular Resampling software on the Xeleris workstation. This reduction in views reduced the idle time from 11 to 8 min.

Unlike in previous studies [11, 15], our patients were administered using constant target activities of 670 MBq per patient instead of weight-dependent activities of 10 MBq/kg. To obtain images more comparable to those used in the previous studies [11, 15], the 5- and 7-s acquisition times per view used in the list-mode resampling were adjusted separately for each patient as if they had received weight-dependent activities of 10 MBq/kg.

For the best possible reproduction of an image set, the 16-min images were processed using the same software and parameters as in the previous study [11]. These images were reconstructed with the Evolution for Bone SPECT software on the Xeleris workstation using the ordered-subset expectation maximization algorithm with 3 iterations and 10 subsets and corrections for photon attenuation and collimator response. A Butterworth post-filter with a cutoff frequency of 0.48 cycles/cm and an order of 1.2 was applied.

The new 32- and 26-min total acquisition time data sets were reconstructed as the original SPECT data using HybridRecon-Oncology software, except the Gaussian filter FWHMs were increased to 10 and 12 mm, respectively. The FWHMs of increased filtering were selected such that the 32- and 26-min images had noise levels similar to those of the original 50-min SPECT images.

### Qualitative image analysis

Qualitative analysis was performed in two rounds. The first round included the originally acquired images, images with 10, 8, 6, and 4% energy window widths, and images with a 256 × 256 matrix from 15 patients. These patients were selected such that the ratio of patients with bone metastases (*n* = 8) to patients with only benign findings (*n* = 7) was similar to that ratio in the original 30-patient population. The second round included the originally acquired 50-min images and images with 38-, 32-, 26-, and 20-min acquisition times from all 30 patients. Lesion visibility and overall image quality were scored by three experienced nuclear medicine physicians on a five-point scale: 1 = insufficient, 2 = almost sufficient, 3 = sufficient, 4 = good, and 5 = excellent for diagnostic use.

### Quantitative image analysis

The originally acquired 50-min images, images with 41-, 32-, 26-, and 20-min acquisition times, images with 10, 8, 6, and 4% energy window widths, and images with 256 × 256 matrix from all 30 patients were included in the quantitative analysis. Benign and metastatic lesions were first segmented from the original images using an initial threshold of SUV = 12. The threshold was lowered if the resulting VOI was clearly smaller than the area of high uptake. The threshold was increased if another high-uptake area was nearby. The same threshold value was used for the same lesion in different images. From the resulting VOIs, lesion mean, maximum, and peak SUVs (SUV_mean_, SUV_max_, SUV_peak_) and volume were measured.

In addition, 5–10 circular regions of interest (ROIs) with a 1-cm diameter were drawn on normal appearing bone adjacent to the lesion. These ROIs were summed to form the background VOI, whose mean SUV (SUV_mean,bg_) and SD of SUV (SUV_SD,bg_) were defined. Contrast was then calculated by dividing the difference between SUV_mean_ and SUV_mean, bg_ by SUV_mean, bg_, noise was calculated by diving SUV_SD, bg_ by SUV_mean, bg_, and the contrast-to-noise ratio (CNR) was calculated by dividing contrast by noise.

### Diagnostic performance analysis

Diagnostic performance analysis included the original 50-min SPECT images and the specially processed 32-, 26-, and 16-min images of all 30 patients. Suspicious bone metastatic lesions were reported from the fused SPECT/CT images by two experienced nuclear medicine physicians. The lesions were reported in a pessimistic manner, such that equivocal lesions were considered metastatic. In addition, overall image quality was scored on the five-point scale described earlier.

To create true positive, true negative, false positive and false negative classes, the reported lesions were validated against the reference diagnosis, which was created during the previous clinical trial. The reference diagnosis is based on the consensus reading of ^99m^Tc-HMDP planar bone scintigraphy and SPECT/CT, ^18^F-PSMA-1007 PET/CT, 1.5-T whole-body diffusion-weighted magnetic resonance, and contrast enhanced CT imaging and clinical, laboratory, and follow-up data [14].

The diagnostic performance of the 50-, 32-, 26-, and 16-min images was compared at the patient, region, and lesion levels. In the region-level analysis, the skeleton was divided into six segments: skull, spine, ribs, pectoral girdle and sternum, pelvis, and limbs.

### Statistical analysis

Statistical analyses were performed using MedCalc statistical software (version 19.2.6, MedCalc Software Ltd, Ostend, Belgium). Lesion visibility and overall image quality scores given by the readers were pooled, reported using the mean and SD, and compared using the Wilcoxon test for paired samples. Lesion visibility and overall image quality failure rates represent the percentage of images rated 1 or 2, i.e., not sufficient for diagnostic use. The failure rates were compared using the *N*–1 chi-squared test.

The median, percentiles, and interquartile range (IQR) are used to describe non-normally distributed data. Differences in SUV measures are reported by Bland–Altman analysis, where the 2.5th and 97.5th percentiles correspond to the 95% limits of agreement (LOA_95%_).

Diagnostic performance was evaluated in terms of sensitivity, specificity, accuracy, and area under the receiver-operating characteristic curve (AUC). The sensitivity, specificity, and accuracy were compared between different images at the patient and region levels using Fisher’s exact test. AUC values were calculated using the trapezoid rule and compared between different images using the method of Hanley and McNeil. *P* values < 0.05 were considered statistically significant.

## Results

### Qualitative image analysis

**Fig. 1:**
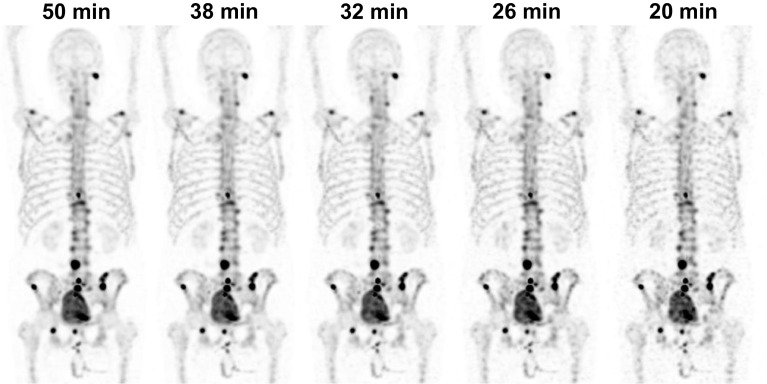
Whole-body ^99m^Tc-HMDP SPECT maximum intensity projections of an 80-year-old prostate cancer patient with different acquisition times. The images are filtered using a Gaussian filter with 7-mm FWHM

Original images scored best in terms of both lesion visibility and image quality. However, the energy window could be narrowed to 8%, the acquisition time reduced to 38 min, and the matrix size increased to 256 × 256 without significantly affecting lesion visibility or image quality failure rates. The overall image quality scores were significantly different between images with 8 and 6% energy windows (*p* = 0.03) and between images with 38 and 32 min acquisition times (*p* < 0.001). The overall image quality failure rate was not significantly different between images with 8 and 6% energy windows, but it was rather high (27–31%). The given scores for lesion visibility and overall image quality in different images and their corresponding failure rates are summarized in Table 1. Figure 1 contains a visual example of how the overall image quality decreases with the acquisition time.

**Table 1:** Scores and failure rates for lesion visibility and image quality with different acquisition parameters

Acquisition time (min)	Energy window width (%)	Matrix size	*N*	Lesion visibility score, mean (SD)	Overall image quality score, mean (SD)	Lesion visibility failure rate	Overall image quality failure rate
50	15	128 × 128	45	3.8 (0.8)	3.6 (0.9)	4%	11%
50	10	128 × 128	45	3.8 (0.9)	3.5 (1.0)	4%	16%
50	8	128 × 128	45	3.7 (1.0)	3.4 (1.1)	11%	27%
50	6	128 × 128	45	3.6 (0.9)	3.1 (1.0)^a^	9%	31% ^a^
50	4	128 × 128	45	3.5 (0.9)^a^	2.7 (1.1)^a^	7%	49% ^a^
50	15	256 × 256	45	3.7 (0.9)	3.3 (1.0)^a^	7%	22%
50	15	128 × 128	90	4.2 (0.8)	3.8 (1.0)	0%	13%
38	15	128 × 128	90	3.8 (0.8)^b^	3.4 (1.0)^b^	2%	19%
32	15	128 × 128	90	3.4 (0.9)^b^	2.9 (1.0)^b^	11%^b^	37%^b^
26	15	128 × 128	90	3.0 (1.0)^b^	2.2 (1.0)^b^	32%^b^	67%^b^
20	15	128 × 128	90	2.3 (1.0)^b^	1.4 (0.6)^b^	58%^b^	92%^b^

*N*  Total number of scores, i.e., the number of readers multiplied by the number of images. ^a ^Statistically significant difference (*p* < 0.05) compared with original images (*N* = 45). ^b ^Statistically significant difference (*p* < 0.05) compared with original images (*N* = 90)

### Quantitative image analysis

A total of 130 lesions were included in the quantitative analysis. The SUV threshold used for lesion segmentation varied from 3 to 15 with a median of 10. Generally, SUV measures and lesion volumes were not affected by changes in energy window width, matrix size or acquisition time per view (Additional file 1). The only exception was noticeably low SUV_peak_ in images with 256 × 256 matrix size, as the median difference was -13% with respect to original images, and LOA_95%_ ranged from −24 to −2%. The median differences for other measures and images ranged from −4 to 2%. SUV_mean_ was the most robust measure, as the width of LOA_95%_ for the difference ranged from 11 to 22 percentage units. The widths of LOA_95%_ for SUV_max_, SUV_peak_, and lesion volume ranged from 25 to 48, 22 to 49, and 61 to 114 percentage units, respectively.

Acquisition time shortening, energy window narrowing, and 256 × 256 image matrix all increased contrast slightly but less than they increased noise, resulting in decreased CNR (Table 2). Energy window narrowing reduced the sensitivity of the SPECT acquisition, such that the conversion factors acquired from the calibration measurement were 87.6, 80.2, 74.3, 65.1, and 49.5 cps/MBq for energy window widths of 15, 10, 8, 6, and 4%, respectively.

**Table 2:** Contrast, noise, and CNR of images with different acquisition parameters

Acquisition time (min)	Energy window width (%)	Matrix size	Contrast, median (IQR)	Noise, median (IQR)	CNR, median (IQR)
50	15	128 × 128	2.0 (1.4–3.2)	0.09 (0.08–0.11)	22.2 (15.7–31.1)
50	10	128 × 128	2.0 (1.4–3.4)	0.12 (0.09–0.16)	17.5 (12.0–24.3)
50	8	128 × 128	2.1 (1.4–3.5)	0.13 (0.10–0.18)	17.4 (12.6–25.8)
50	6	128 × 128	2.2 (1.4–3.5)	0.15 (0.12–0.18)	14.4 (10.4–21.3)
50	4	128 × 128	2.3 (1.4–3.6)	0.18 (0.15–0.22)	12.8 (8.2–20.1)
50	15	256 × 256	2.1 (1.4–3.2)	0.12 (0.10–0.15)	17.1 (12.1–24.5)
41	15	128 × 128	2.0 (1.4–3.3)	0.12 (0.10–0.16)	18.0 (12.0–25.8)
32	15	128 × 128	2.1 (1.4–3.3)	0.15 (0.11–0.19)	14.0 (9.4–20.3)
26	15	128 × 128	2.1 (1.4–3.3)	0.17 (0.14–0.23)	12.7 (8.3–19.7)
20	15	128 × 128	2.2 (1.5–3.6)	0.22 (0.18–0.29)	10.8 (7.1–15.3)

### Diagnostic performance analysis

According to the reference diagnosis, 12 patients out of 30 had bone metastases, 35 different skeletal regions were metastatic, and altogether 100 lesions were considered positive for bone metastases. All metastatic patients were detectable, but 10 metastatic bone regions and 28 bone metastases could not be detected by original SPECT/CT analysis.

Acquisition time reduction had little effect on the diagnostic performance, as sensitivity, specificity, accuracy, and AUC were not significantly different between the 50-min total acquisition time and reduced acquisition time images. The average patient-level sensitivities of the two readers were 88, 92, 100, and 96% for the 50-, 32-, 26-, and 16-min images, respectively, and the corresponding specificities were 78, 84, 84, and 78%. The average region-level sensitivities of the two readers were 55, 58, 59, and 56% for the 50-, 32-, 26-, and 16-min images, respectively, and the corresponding specificities were 95, 98, 96, and 95%. The number of equivocal lesions tended to increase as the acquisition time decreased. The results of the patient-, region-, and lesion-level analyses with decreasing acquisition time are given in Tables 3, 4, and 5.

**Table 3:** Patient-level analysis with decreasing acquisition time

	Acquisition time (min)	Sensitivity (95% CI)	Specificity (95% CI)	Accuracy (95% CI)	AUC (95% CI)
Reader A	50	83% (52–98%)	83% (59–96%)	83% (65–94%)	0.83 (0.65–0.94)
	32	83% (52–98%)	78% (52–94%)	80% (61–92%)	0.81 (0.62–0.93)
	26	100% (74–100%)	78% (52–94%)	87% (69–96%)	0.89 (0.72–0.97)
	16	100% (74–100%)	72% (47–90%)	83% (65–94%)	0.86 (0.69–0.96)
Reader B	50	92% (62–100%)	72% (47–90%)	80% (61–92%)	0.82 (0.64–0.94)
	32	100% (74–100%)	89% (65–99%)	93% (78–99%)	0.94 (0.80–1.00)
	26	100% (74–100%)	89% (65–99%)	93% (78–99%)	0.94 (0.80–1.00)
	16	92% (62–100%)	83% (59–96%)	87% (69–96%)	0.88 (0.70–0.97)

**Table 4:** Region-level analysis with decreasing acquisition time

	Acquisition time (min)	Sensitivity (95% CI)	Specificity (95% CI)	Accuracy (95% CI)	AUC (95% CI)
Reader A	50	57% (39–74%)	96% (91–98%)	88% (83–93%)	0.77 (0.70–0.83)
	32	57% (39–74%)	97% (92–99%)	89% (83–93%)	0.77 (0.70–0.83)
	26	63% (45–79%)	94% (89–98%)	88% (83–93%)	0.79 (0.72–0.84)
	16	57% (39–74%)	91% (85–95%)	84% (78–89%)	0.74 (0.67–0.80)
Reader B	50	53% (36–69%)	94% (88–97%)	85% (79–90%)	0.77 (0.70–0.83)
	32	58% (41–74%)	98% (94–100%)	89% (84–94%)	0.80 (0.74–0.86)
	26	55% (38–71%)	98% (94–100%)	89% (84–93%)	0.79 (0.72–0.85)
	16	55% (38–71%)	98% (94–100%)	89% (83–93%)	0.79 (0.72–0.85)

**Table 5:** Lesion-level analysis with decreasing acquisition time

	Acquisition time (min)	Number of positive lesions reported	Number of true positive lesions	Number of false positive lesions	Number of false negative lesions	Number of equivocal lesions reported	Ratio of equivocal to all detected lesions (%)
Reader A	50	52	49	3	51	11	17
	32	40	36	4	64	14	26
	26	37	35	2	65	17	31
	16	49	43	6	57	26	35
Reader B	50	55	52	3	48	7	11
	32	54	53	1	47	5	8
	26	51	49	2	51	3	6
	16	52	50	2	50	4	7
Total	50	107	101	6	99	18	14
	32	93	89	5	111	19	17
	26	88	84	4	116	20	19
	16	101	93	8	107	30	23

**Fig. 2:**
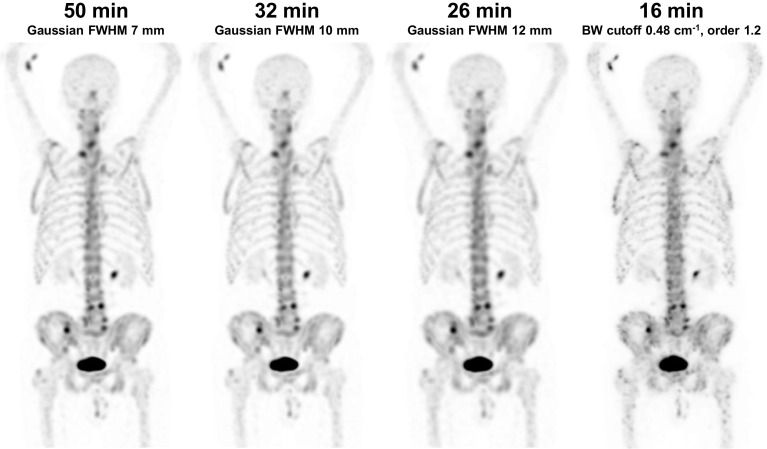
Whole-body ^99m^Tc-HMDP SPECT maximum intensity projections of a 72-year-old prostate cancer patient with different acquisition times and post-processing filters. The 50-, 32-, and 26-min images are filtered using Gaussian filters with FWHMs of 7, 10, and 12 mm, respectively, and the 16-min image is filtered using a Butterworth filter with a cutoff frequency of 0.48 cycles/cm and an order of 1.2. The 16-min image is acquired and processed using the same parameters as in an earlier study [11]

Even though noise was suppressed by widening the Gaussian filter, the overall image quality scores were still lower in the images with shorter acquisition times. The mean (SD) image quality scores were 3.4 (1.0), 2.9 (0.7), 2.7 (0.7), and 1.8 (0.7), and the image quality failure rates were 20, 32, 45, and 85% for 50-, 32-, 26-, and 16-min images, respectively. Examples of images with different acquisition times and filters are shown in Fig. 2.

## Discussion

The most common current approach to diagnose prostate cancer bone metastases is still planar bone scintigraphy and CT separately. Acquisition time is an important factor regarding the feasibility of whole-body bone SPECT/CT for the imaging of bone metastases. With a shorter acquisition time, the clinical use could potentially increase significantly. Furthermore, whole-body bone SPECT/CT has shown superior diagnostic performance compared to planar bone scintigraphy [1–6]. However, the breakthrough of bone SPECT/CT into clinical routine has yet to become [8].

To the best of our knowledge, this is the first receiver-operating characteristic analysis of fast whole-body bone SPECT/CT in actual diagnostic use with a multimodal reference standard. Previously, fast bone SPECT has been investigated using various approaches. Gregoire et al. evaluated visual image quality [11]. Alqahtani et al. optimized reconstruction parameters to preserve image quality with reduced acquisition time [16]. Zacho et al. demonstrated fast partial-body bone SPECT/CT as an add-on to whole-body planar bone scintigraphy [15, 17]. Ichikawa et al. [18] presented fast bone SPECT by using a custom-designed phantom and a reconstruction algorithm based on CT zonal mapping. Pan et al. [19] proved the feasibility of deep learning for enhancing low-count bone SPECT data. In addition, the physical performance of a CZT system similar to ours has been described by Ito et al. [20]. The general-purpose CZT system has been used to reduce examination times in bone [11], myocardial perfusion [21], and dopamine transporter imaging [22].

We evaluated the effects of fast SPECT acquisition on the diagnostic performance of whole-body ^99m^Tc-HMDP SPECT/CT and showed that the total acquisition time can be reduced from 50 to even 16 min without any loss of diagnostic performance. Patient- and region-level sensitivity, specificity, accuracy, and AUC values for bone metastasis detection were not significantly different between the 50-min images and any of the shorter time images.

No systematic changes could be identified for diagnostic performance values either on the patient or region level with shortening acquisition time. The only identified systematic change was the increase in equivocal lesions for one reader when the acquisition time became shorter. The higher number of equivocal lesions was probably caused by increased noise and decreased image quality. However, the number of equivocal lesions might become lower as readers gain more experience on noisier short-acquisition-time images.

According to the quantitative and qualitative analyses, a noise level of approximately 0.10 was associated with generally accepted image quality. This noise level was also used as the target when selecting filters for the 32- and 26-min images used in the diagnostic performance analysis. However, the overall image quality of these images was still evaluated to be lower than that of 50-min images. The 16-min images were processed differently from other images to mimic the processing method used in a previous study [11]. The short acquisition time combined with unoptimized image processing resulted in the highest number of equivocal lesions but had little effect on the patient- and region-level diagnostic performance. Diagnostic performance being unaffected by the acquisition time was most likely caused by the preserved high lesion contrast in the images with short acquisition times (Table 2). The reconstruction parameters of the 50-, 32-, and 26-min images were similar to those suggested to be optimal by Alqahtani et al. [16], except post-processing filtering was increased for the 32- and 26-min images.

Even though the 16-min SPECT/CT images resulted high for metastasis detection, most readers considered that image quality was insufficient for diagnostic use. However, visually evaluated image quality can be very reader-dependent, as images similar to our 16-min images have been rated sufficient for diagnostic use in a previous study [11]. Generally, visual image quality grades given by the reading physicians may partly reflect the image quality to which they are accustomed.

In line with a recent study [23], the results of SUV and lesion volume measurements were not affected by changes in the acquisition parameters. SUV_peak_ with a 256 × 256 matrix was the only exception, but this can be explained by the difference in voxel size between 128 × 128 and 256 × 256 matrices, which causes different actual volumes for the 1 cm^3^ cube used for measuring SUV_peak_. Moreover, the repeatability of SUV and lesion volume measurements is expected to decrease as image noise increases [23].

Energy window narrowing and a larger image matrix size increased quantitatively measured contrast, but this relatively small change did not affect qualitative lesion visibility scores. Noise increased more than contrast, resulting in decreased CNR and overall image quality scores. However, the overall image quality failure rates were not significantly higher when the energy window was narrowed to 8%, the acquisition time was reduced to 38 min, or the matrix size increased to 256 × 256. Additionally, the contrast of the smallest lesions did not increase significantly by increasing the image matrix size. To properly benefit from the 256 × 256 matrix, more advanced reconstruction algorithms, such as those using CT for anatomical a priori information [24, 25], are likely required.

In the reconstruction, we employed a rather sophisticated scatter correction method based on the CT attenuation map and Monte Carlo simulation. If the scatter correction had been omitted, the contrast increase in narrowed energy window images might have been more apparent. The noise increase in narrow energy window images is mostly caused by reduced counts, but it may also be associated with detector uniformity. We used a single uniformity map acquired with a 15% energy window for all energy windows, although it would have been more suitable to acquire separate uniformity maps for different energy windows [26]. However, the post-acquisition change of the uniformity map was not supported by the list-mode resampling software at that time. Another improvement would be the modeling of the characteristic hole tailing effect of CZT detectors during the reconstruction [27].

We used only symmetric energy windows, but it might have been beneficial to explore asymmetric energy windows where only the lower threshold is adjusted, as scattered photons are more likely included in the lower end of the accepted energy spectrum. The asymmetric energy window has also been shown to slightly improve image quality in planar bone scintigraphy [28]. Although we could not find benefits from the energy window narrowing in the current study, it should be noted that our focus was on bone SPECT images, where the lesions are more active than the background, as opposed to, for example, cardiac SPECT, where the lesions are less active than the background. Under those conditions, narrowing the energy window might have a different effect on image quality, as scatter correction has been shown to increase cold contrast slightly more than hot contrast [29]. On the other hand, it has been reported that the contrast increase caused by scatter correction is reduced when the object size decreases and that scatter correction could even decrease the contrast of very small (diameter ≤ 6 mm) objects [29].

Regarding the future of skeletal imaging in nuclear medicine, we expect a shift from planar bone scintigraphy to whole-body SPECT/CT [6]. In this development, the reduction of acquisition time for whole-body SPECT/CT is of paramount importance. Currently, the acquisition time for whole-body SPECT/CT examinations is typically more than 40 min, and for planar bone scintigraphy, it is approximately 20 min. In this study, we have shown that the acquisition time of whole-body SPECT can be lowered from 50 to 16 min without losing diagnostic performance for lesion detection. To smoothen the transition from planar bone scintigraphy to whole-body SPECT/CT, we have validated reprojected bone SPECT/CT as a method to facilitate the reading of SPECT images [30].

This study was performed using a digital CZT SPECT/CT system. However, the results of acquisition time shortening can be generalized to analogic SPECT/CT systems by considering the differences in system sensitivity and spatial resolution. The acquisition time can be normalized with respect to the sensitivity difference between the SPECT/CT systems if they have similar spatial resolution. The volumetric sensitivity of our digital CZT SPECT/CT system is 364 kcps/(MBq/cm^3^) with a 20% energy window width, and the system spatial resolution (FWHM) is 3.8–5.4 mm when no post-processing filtering is applied [31].

The limitations of our study include a rather low number of patients and only two readers for the evaluation of diagnostic performance. Ideally, the images with different acquisition times would have been read by different physicians. However, the order in which the image sets were read was from the shortest acquisition time to the longest, and hence, no positive bias is expected for the diagnostic performance of the 16-min images. Additionally, there were at least three weeks between the readings of different images from the same patient.

We validated fast whole-body bone SPECT/CT in prostate cancer patients. The high osteogenic features of prostate cancer may have promoted our findings [32], so further research is required to generalize our results into other cancers. This is important, as the use of bone SPECT/CT in the diagnosis of prostate cancer may decline due to the increased use of PSMA PET and SPECT ligands in the near future, which will allow for the detection of both bone and soft tissue metastases [33, 34].

## Conclusion

Whole-body ^99m^Tc-HMDP SPECT/CT can be acquired using a general-purpose CZT system in less than 20 min without any loss in diagnostic performance in metastasis staging of high-risk prostate cancer patients.

## Supplementary Information

**Additional file 1**. SUV_mean_, SUV_max_, SUV_peak_, and lesion volume in images with different acquisition parameters.

## Data Availability

The anonymized datasets analyzed during the current study are available from the corresponding author on a reasonable request.

## Abbreviations

AUC: Area under receiver-operating characteristic curve
CI: Confidence interval
CNR: Contrast-to-noise ratio
CT: X-ray computed tomography
CZT: Cadmium zinc telluride
FWHM: Full width at half maximum
HMDP: Hydroxymethylene diphosphonate
IQR: Interquartile range
LOA: Limits of agreement
PET: Positron emission tomography
PSMA: Prostate-specific membrane antigen
ROI: Region of interest
SD: Standard deviation
SPECT: Single-photon emission computed tomography
SUV: Standardized uptake value
VOI: Volume of interest
