# Supplementary material for: Detection of prostate cancer bone metastases with fast whole-body 99mTc-HMDP SPECT/CT using a general-purpose CZT system
Source: EJNMMI Phys. 2022 Dec 12;9:85. doi: 10.1186/s40658-022-00517-4 (PMC9743860; doi:10.1186/s40658-022-00517-4)
Supplement: Supplementary file 1 — Additional file 1. SUVmean, SUVmax, SUVpeak, and lesion volume in images with different acquisition parameters. [file 40658_2022_517_MOESM1_ESM.docx]

**Additional file 1.** SUV_mean_, SUV_max_, SUV_peak_, and lesion volume in images with different acquisition parameters.

| Acquisition time | Energy window  width | Matrix size | SUV_mean_ median (IQR) | SUV_max_ median (IQR) | SUV_peak_ median (IQR) | Lesion volume (ml) median (IQR) |
| --- | --- | --- | --- | --- | --- | --- |
| 50 min | 15% | 128 x 128 | 14.3 (11.4–16.4) | 19.0 (14.5–29.6) | 16.0 (12.5–23.7) | 2.5 (1.6–4.0) |
| 50 min | 10% | 128 x 128 | 14.2 (11.7–16.7) | 19.0 (14.8–29.5) | 16.1 (12.3–23.9) | 2.4 (1.6–4.1) |
| 50 min | 8% | 128 x 128 | 14.3 (11.3–16.5) | 20.0 (14.4–29.6) | 16.5 (12.2–23.7) | 2.5 (1.6–3.9) |
| 50 min | 6% | 128 x 128 | 14.4 (11.7–16.9) | 20.3 (14.5–29.8) | 16.5 (12.4–23.0) | 2.4 (1.6–3.9) |
| 50 min | 4% | 128 x 128 | 14.2 (11.5–16.8) | 19.9 (14.8–30.6) | 16.6 (12.1–23.7) | 2.4 (1.5–3.9) |
| 50 min | 15% | 256 x 256 | 14.0 (11.3–16.3) | 19.3 (14.7–28.9) | 14.4 (10.7–20.3) | 2.3 (1.5–3.8) |
| 41 min | 15% | 128 x 128 | 14.0 (11.7–16.7) | 18.6 (14.5–29.1) | 15.9 (12.3–23.3) | 2.4 (1.6–4.0) |
| 32 min | 15% | 128 x 128 | 14.0 (11.3–16.8) | 18.9 (14.4–28.1) | 15.8 (12.4–23.0) | 2.5 (1.6–4.0) |
| 26 min | 15% | 128 x 128 | 14.1 (11.5–16.7) | 18.7 (14.6–28.9) | 15.9 (12.6–23.8) | 2.5 (1.6–3.9) |
| 20 min | 15% | 128 x 128 | 14.2 (11.6–16.5) | 19.3 (14.7–29.4) | 16.5 (12.6–23.3) | 2.6 (1.5–4.1) |
